# Leukemia inhibitory factor modulates the peripheral immune response in a rat model of emergent large vessel occlusion

**DOI:** 10.1186/s12974-018-1326-y

**Published:** 2018-10-15

**Authors:** Stephanie M. Davis, Lisa A. Collier, Edric D. Winford, Christopher C. Leonardo, Craig T. Ajmo, Elspeth A. Foran, Timothy J. Kopper, John C. Gensel, Keith R. Pennypacker

**Affiliations:** 1 Department of Neurology, University of Kentucky, 741 S. Limestone BBSRB B457, Lexington, KY 40536-0905 USA; 2 Department of Neuroscience, University of Kentucky, 800 Rose St. Lexington, Lexington, KY 40536 USA; 3 Department of Physiology, University of Kentucky, 800 Rose St. MS508, Lexington, KY 40536 USA; 4 Spinal Cord and Brain Injury Repair Center, University of Kentucky, 741 S. Limestone BBSRB B463, Lexington, KY 40536 USA; 5 Department of Molecular Pharmacology and Physiology, University of South Florida, 12901 Bruce B. Downs Blvd MDC 8, Tampa, FL 33612 USA; 6 Department of Molecular Medicine, University of South Florida, 12901 Bruce B. Downs Blvd MDC 7, Tampa, FL 33612 USA

**Keywords:** Stroke, Inflammation, Macrophages, Ischemia, Cytokines

## Abstract

**Background:**

The migration of peripheral immune cells and splenocytes to the ischemic brain is one of the major causes of delayed neuroinflammation after permanent large vessel stroke. Other groups have demonstrated that leukemia inhibitory factor (LIF), a cytokine that promotes neural cell survival through upregulation of antioxidant enzymes, promotes an anti-inflammatory phenotype in several types of immune cells. The goal of this study was to determine whether LIF treatment modulates the peripheral immune response after stroke.

**Methods:**

Young male (3 month) Sprague-Dawley rats underwent sham surgery or permanent middle cerebral artery occlusion (MCAO). Animals were administered LIF (125 μg/kg) or PBS at 6, 24, and 48 h prior to euthanization at 72 h. Bone marrow-derived macrophages were treated with LIF (20 ng/ml) or PBS after stimulation with interferon gamma + LPS. Western blot was used to measure protein levels of CD11b, IL-12, interferon inducible protein-10, CD3, and the LIF receptor in spleen and brain tissue. ELISA was used to measure IL-10, IL-12, and interferon gamma. Isolectin was used to label activated immune cells in brain tissue sections. Statistical analysis was performed using one-way ANOVA and Student’s *t* test. A Kruskal-Wallis test followed by Bonferroni-corrected Mann-Whitney tests was performed if data did not pass the D’Agostino-Pearson normality test.

**Results:**

LIF-treated rats showed significantly lower levels of the LIF receptor and interferon gamma in the spleen and CD11b levels in the brain compared to their PBS-treated counterparts. Fluorescence from isolectin-binding immune cells was more prominent in the ipsilateral cortex and striatum after PBS treatment compared to LIF treatment. MCAO + LIF significantly decreased splenic levels of CD11b and CD3 compared to sham surgery. MCAO + PBS treatment significantly elevated splenic levels of interferon inducible protein-10 at 72 h after MCAO, while LIF treatment after MCAO returned interferon inducible protein 10 to sham levels. LIF administration with interferon gamma + LPS significantly reduced the IL-12/IL-10 production ratio compared to macrophages treated with interferon gamma + LPS alone.

**Conclusions:**

These data demonstrate that LIF promotes anti-inflammatory signaling through alterations of the IL-12/interferon gamma/interferon inducible protein 10 pathway.

## Background

Permanent occlusion of the brain’s major arteries, also known as emergent large vessel occlusion (ELVO), is one of the deadliest types of acute ischemic stroke and a major cause of adult disability [1–3]. Due to the size of the thrombus creating the blockage, ELVO patients are often resistant to treatment with tissue plasminogen activator (tPA), the only FDA-approved drug for the treatment of stroke. The increased use of stent retrievers for performing endovascular thrombectomy has allowed for restoration of cerebral blood flow up to 24 h after the onset of symptoms [4–6]. However, patients who are deemed ineligible for endovascular thrombectomy are left without sufficient treatment options [7]. Our laboratory has examined the inflammatory response to stroke using the permanent middle cerebral artery occlusion, which simulates ELVO.

Although neural cell death during large-vessel stroke is commonly associated with energy failure and low levels of oxygen, resulting brain damage occurs during two distinct phases of cellular damage. The acute cytotoxicity phase, which occurs minutes to hours after the onset of stroke, results in the death of cells within the ischemic core. The second phase of brain damage begins approximately 12–24 h after the onset of focal cerebral ischemia and results from systemic activation of the peripheral immune system [8, 9]. Within the ischemic brain, dying neurons and glia release compounds such as ATP, fractalkine, or other danger-associated molecular patterns, which activate microglia, the resident macrophage-like immune cells in the brain [10–13]. Once activated, microglia damage the blood-brain barrier via the release of pro-inflammatory cytokines and matrix metalloproteinases [14].

Increased permeability of the blood-brain barrier contributes to cerebral edema and renders cells in the parenchyma vulnerable to invading peripheral immune cells. These immune cells infiltrate the brain approximately 48 h after cerebral ischemia and include monocytes/macrophages, lymphocytes, and neutrophils [15–17]. Although certain populations of these immune cells, such as anti-inflammatory macrophages/microglia, may play a crucial role in phagocytosis and tissue repair, pro-inflammatory leukocytes potentiate further damage in the area surrounding the ischemic core (penumbra). In addition, peripheral B and T cells that are exposed to CNS-specific antigens may contribute towards the post-stroke adaptive immune response by antibody secretion or direct cytotoxicity [18, 19]. This secondary phase continues until approximately 96 h after the onset of stroke, when the infarct reaches its maximum volume [20, 21].

Of the peripheral immune cell populations that migrate to the brain after stroke, a substantial portion of them originate in the spleen. After stroke, elevated levels of norepinephrine and epinephrine mediate splenic contraction through the activation of α- and β-adrenoreceptors in the white pulp, where splenic leukocytes reside [22, 23]. These leukocytes are released into the peripheral circulation, although the secretion of pro-inflammatory mediators causes these cells to migrate towards the injured hemisphere and amplify the inflammatory response in the brain. Previous studies revealed inverse relationships between post-stroke spleen weights and infract volumes in addition to spleen weights and splenic CD8+ cytotoxic T cell count. These findings show that greater splenic atrophy is the result of more infiltrating immune cells, which mediate delayed neurodegeneration [24–27]. Our lab previously demonstrated that splenectomy 2 weeks prior to permanent middle cerebral artery occlusion (MCAO) had significantly increased infarct volumes after stroke [26]. Interferon gamma (IFNγ) production by T cells and natural killer (NK) cells and subsequent induction of interferon inducible protein 10 (IP-10) triggers the first step in the delayed inflammatory response [26, 28–30]. Clinical data have shown that this post-stroke splenic response not only occurs in human stroke patients, but also differs among patients of different ages and racial backgrounds [31–33].

Leukemia inhibitory factor (LIF) is a cytokine in the IL-6 family that promotes survival of neurons and glia in several animal models of neurodegenerative disease, such as amyotrophic lateral sclerosis [34, 35], multiple sclerosis [36–42], spinal cord injury [43, 44], and stroke [45–47]. Our laboratory has identified several cytoprotective mechanisms of LIF, which include the upregulation of antioxidant enzymes peroxiredoxin IV and metallothionein III in oligodendrocytes [46, 48] and superoxide dismutase 3 in neurons [47]. Other groups have shown that in the efficacy of LIF in treating animal models of neurodegenerative disease may lie in its ability to modulate the immune system in addition to its pro-survival signaling [42, 49].

Several groups have reported that LIF alters the phenotype of macrophages/microglia from a pro-inflammatory to an anti-inflammatory phenotype. While pro-inflammatory macrophages worsen tissue damage during stroke via the release of pro-inflammatory mediators (tumor necrosis factor-α, IL-12, IL-6, IL-1β, nitric oxide, etc.), anti-inflammatory cells are primarily phagocytic and release anti-inflammatory mediators. In addition, these macrophages/microglia recruit other anti-inflammatory cells such as CD4 + CCR4+ helper T lymphocytes and regulatory T cells (Tregs) to the site of injury. Duluc et al. demonstrated that LIF and IL-6, in conjunction with macrophage colony-stimulating factor cause monocytes to differentiate into IL-12^low^ IL-10^high^ tumor-associated macrophages, a specialized class of anti-inflammatory leukocytes that protect cancerous growths from NK cells [50]. Although pro-inflammatory macrophages/microglia further damage neural cells after stroke by perpetuating inflammation and producing reactive oxygen species, macrophages/microglia with an anti-inflammatory phenotype contribute to the repair process during ischemia [13, 51]. Therefore, LIF may indirectly reduce the neuroinflammation-associated damage during stroke by increasing the population of anti-inflammatory macrophages/microglia.

Previously, we showed that LIF reduces tissue damage and improves function recovery after stroke through the Akt-dependent upregulation of antioxidant enzymes in neurons and oligodendrocytes [46, 47]. The purpose of this study is to determine whether LIF alters the post-stroke splenic response by changing the phenotype of macrophages/microglia from a pro-inflammatory to an anti-inflammatory phenotype.

## Methods

### Animal care

Animal procedures were pre-approved by the Institutional Animal Care and Use Committee at the University of South Florida and performed according to the NIH Guide for the Care and Use of Laboratory Animals. Power analysis was used to determine the appropriate number of animals for each experiment. Sprague–Dawley rats were purchased from Envigo (Indianapolis, IN, USA). C57BL/6 mice were purchased from Jackson Laboratories (Bar Harbor, ME, USA). Animals were maintained on a 12-h light–dark cycle (07:00–19:00 h) in a climate-controlled room and allowed access to food and water ad libitum. In vivo procedures were performed on young (3 months), male rats weighing between 300 and 350 g. For in vitro experiments, bone marrow was harvested from young mice (2–3 months).

### Middle cerebral artery occlusion

Permanent focal cerebral ischemia was induced using the intraluminal middle cerebral artery occlusion (MCAO) model as previously described [52]. A 40-mm monofilament was introduced through the ligated external carotid artery and advanced through the internal carotid artery. The filament was advanced until it reached the origin of the middle cerebral artery. Reduction in cerebral blood flow was confirmed using Laser Doppler (Moore Lab Instruments, Farmington, CT). Only animals experiencing ≥ 60% reduction in cerebral perfusion were included in the study. Animals subjected to the sham MCAO procedure were anesthetized and underwent exposure of the common carotid artery without subsequent occlusion of the middle cerebral artery.

### Drug treatment

All animals were treated prophylactically with ketoprofen (10 mg/kg s.c.), atropine (0.25 mg/kg s.c.) with two additional doses of ketoprofen at 24 and 48 h post-MCAO. Recombinant human LIF (ProSpec, Ness Ziona, Israel) (125 μg/kg) or PBS (pH 7.4) was administered intravenously at 6, 24, and 48 h post-MCAO as previously described [46, 47]. Animals were randomly assigned to treatment groups and all lab personnel administering drugs were blinded to treatments.

### Tissue collection

Rats were euthanized at 72 h post-MCAO via intraperitoneal injection of ketamine/xylazine solution (75 mg/kg and 7.5 mg/kg) [53]. Spleens were collected immediately prior to perfusion. Animals used for immunohistochemical analysis were perfused transcardially with normal saline followed by 4% paraformaldehyde in phosphate buffer. Animals used for biochemical analysis were perfused with saline but not paraformaldehyde prior to obtaining tissue. Fresh brain tissue was separated into ipsilateral and contralateral hemispheres, snap-frozen, and stored at − 80 °C until further processing. Fixed brains were cryopreserved in 20% followed by 30% sucrose solutions and cut into 30-μm sections using a cryostat. Brain tissue used in these experiments was located between + 1.7 and − 3.3 mm from the bregma.

### Tissue homogenization

To obtain whole cell extracts, frozen tissue was homogenized in whole cell lysis buffer containing the following: 50 mM Tris pH 8, 150 mM NaCl, 0.1% SDS, 1% IGEPAL, 1 mM PMSF, and a Complete Mini protease inhibitor cocktail (Roche Diagnostics, Indianapolis, IN). An electric homogenizer was used to disrupt tissue, and lysates were incubated on ice for 20 min. Tissue lysates were vortexed and pipetted to break up nuclei. Protein concentrations were determined by performing a Bradford Assay according to the manufacturer’s protocol (Bio-Rad, Hercules, CA). Briefly, Bradford reagent containing Coomassie blue was added to diluted protein samples and absorbance was read at 595 nm using a SmartSpec 3000 spectrophotometer (Bio-Rad). Concentrations were determined by comparing the absorbance readings against a standard curve.

### Isolectin staining

Isolectin IB4 (0.05% *v*/*v*) from *Griffonia simplicifolia* conjugated to AlexaFluor® 488 dye was used to label activated macrophages and microglia in the cortical and striatal tissue of PBS- and LIF-treated rat brains according to a previously described procedure [20]. Coverslips were mounted onto slides using VECTASHIELD® medium containing 4′, 6-diamidino-2-phenylindole (DAPI) (Vector Labs, Burlingame, CA). Images were captured using a Nikon Eclipse Ti microscope (Minato, Tokyo, Japan) interfaced with NIS Elements Imaging Software (Nikon).

### 3,3-Diaminobenzidine immunohistochemistry

To detect CD11b-positive cells in brain tissue, 3,3-diaminobenzidine (DAB) immunohistochemistry was performed according to a previously described protocol [54]. The following antibodies were used: mouse α-CD11b (OX42) (1:3000; Bio-Rad; Hercules, CA) and horse α-mouse (1:300; Vector-Labs; Berlingame, CA). Slides were cover slipped with DPX medium (BDH Laboratories, Poole, England) and images were acquired with a Nikon Eclipse T*i* microscope interfaced with NIS Elements Imaging Software (Nikon).

DAB staining of spleen tissue was also performed according to a previously described protocol, albeit with minor modifications. Briefly, cryopreserved spleen tissue sections (30 μm) were dried at 37 °C, rehydrated with PBS (pH 7.4), and permeabilized for 1 h containing 10% goat serum, 0.3% 1 M Lysine, and 0.3% Triton-X-100. Following permeabilization, spleen sections were treated for 40 min with 3% H_2_O_2_ to quench endogenous peroxidase activity. Sections were incubated overnight in the following antibodies: rabbit α-LIFR (1:200; Santa Cruz; RRID:AB_2136015) and mouse α-FoxP3 (1:5000; Abcam; RRID:AB_447114). Secondary detection was achieved using goat α-rabbit and goat α-mouse biotinylated secondary antibodies (1:300; Vector Labs). Slides were mounted with DPX medium (BDH Laboratories) after dehydration with ethanol and clearing with xylenes. All images were acquired using a Nikon Eclipse T*i* microscope interfaced with NIS Elements Imaging Software (Nikon).

### Western blot analysis

Western blotting was used for semi-quantitative measurement of protein expression using a previously described procedure [47]. Briefly, whole cell lysates from brain and spleen tissue were run on 10% SDS-PAGE gels and transferred to nitrocellulose membranes. Membranes were blocked in Li-Cor TBS-based Blocking Buffer (Lincoln, NE) and probed with the following antibodies: rabbit α-LIFR (1:100; Santa Cruz), rabbit α-IL-12 p40 (1:100; Abbiotec RRID:AB_10636335), mouse α-CD11b (1:1000; Abcam). Membranes were incubated in IRDye 800CW goat α-rabbit antibodies (1:20,000; Li-Cor; RRID:AB_2651127) for detection of protein bands. Membranes were visualized using the Odyssey CLx Imaging System (Li-Cor). To normalize for loading, membranes containing whole cell extracts were re-probed with mouse α-β-actin (1:5000; Novus Biologicals; RRID:AB_1216153) and IRDye 680RD goat α-mouse antibodies (1:20,000; Li-Cor; RRID:AB_10956588).

### Bone marrow-derived macrophage cell culture

Bone marrow-derived macrophages (BMDMs) were isolated from C57BL/6 mice (3 months of age) as previously described [55–57]. Briefly, cells were extracted from the femur and tibia and seeded at a density of 8 × 10^5^–1 × 10^6^ cells/ml in Dulbecco’s modified Eagle’s medium (DMEM) containing 10% FBS, 1% penicillin/streptomycin, 1% HEPES, 0.001% β-mercaptoethanol, and 20% supernatant containing macrophage colony-stimulating factor from SL929 cells (gifted by Phillip Popovich from The Ohio State University) [58]. Following 7 days of in vitro differentiation, cells were re-seeded at a density of 1 × 10^6^ cells/ml in DMEM containing 10% FBS, 1% penicillin/streptomycin, 1% HEPES, and 0.001% β-mercaptoethanol. The next day*,* a classically activated phenotype (M1) was induced using N2A medium containing LPS (50 ng/ml) and IFNγ (20 ng/ml). LIF (20 ng/ml) or PBS was co-administered with the LPS and IFNγ. Macrophage-conditioned media was collected 24 h after stimulation and centrifuged at 13,000 rpm at 4 °C for 10 min prior to measurement of IL-12 p40 and IL-10 via ELISA [59].

### ELISA

To measure the release of IFNγ, TNFα, IL-1β, IL-6, and IL-10 in rat spleen tissue, ELISA was performed according to manufacturer’s protocol using the appropriate DuoSet ELISA kits (R&D Systems, Inc., Minneapolis, MN). ELISA was also used to measure IL-12 p40 and IL-10 release in macrophage supernatants according to the manufacturer’s protocol using the Mouse IL-12 p40 and IL-10 ELISA kits (Cat # EMIL12P40 and Cat # EM2IL105; Thermo Fisher Scientific, Waltham, MA).

### Data analysis

Images were minimally processed in a uniform matter across treatment groups and were analyzed using ImageJ software (NIH, Bethesda, MD). The D’Agostino-Pearson test was performed to determine whether data was normally distributed. Statistical analysis for experiments containing two groups was performed using Student’s *t* test, or the Mann-Whitney *U* Test if the data sets did not pass the D’Agostino-Pearson test. Welch’s correction was used in the case of unequal variances. Statistical analysis for experiments containing three or more groups was performed using the one-way ANOVA followed by Fisher’s Protected LSD test to determine individual differences If data did not pass the D’Agostino-Pearson test, the Kruskal-Wallis *H* test was used, and individual differences were detected using Bonferroni-corrected Mann-Whitney *U* tests. A *p* value equal to 0.05 or less was considered statistically significant. All reported *p* values are one-tailed.

## Results

### LIF decreases LIFR expression but does not alter spleen weight after MCAO

**Fig. 1:**
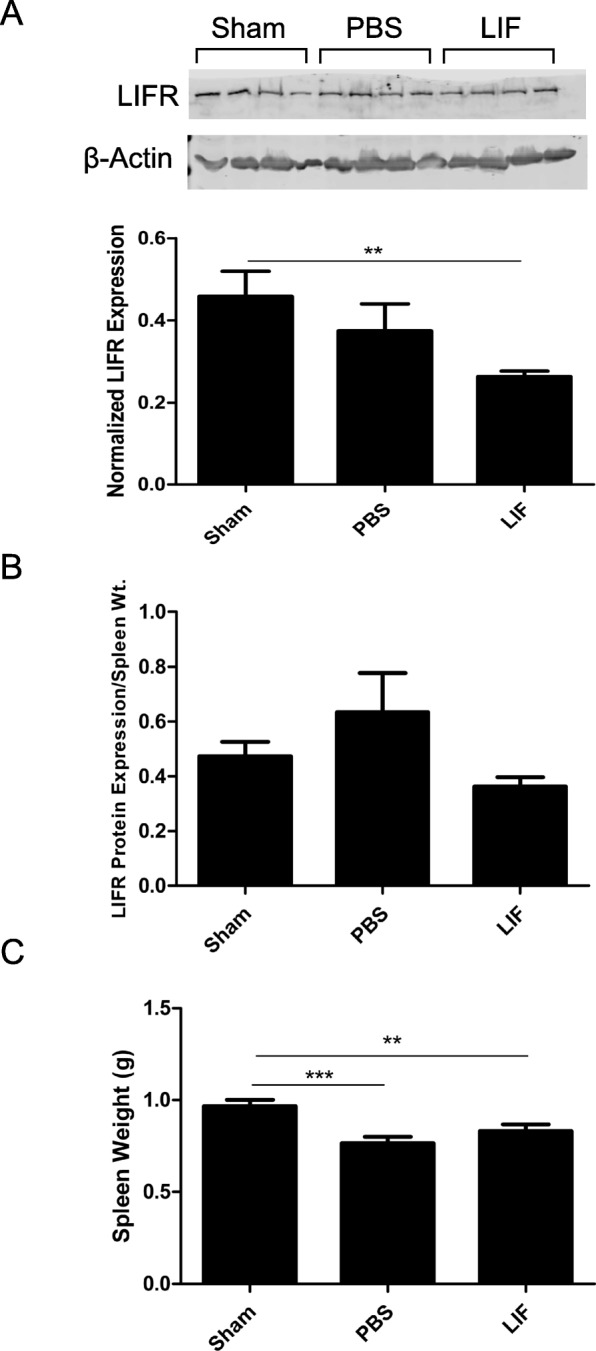
LIF treatment decreases splenic LIFR but does not change spleen weight. **a** Western blotting was used to measure levels of the LIF receptor (LIFR) in the spleen 72 h after sham surgery and MCAO. There was a trend towards decreased LIFR levels after MCAO and a significant decrease in LIFR after MCAO and treatment with LIF (**p < 0.01). *n* = 8 animals per treatment group. **b** When LIFR protein levels were normalized to the spleen weight, there was a trend towards increased normalized LIFR in the PBS-treated group, but this increase was not significant. **c** At 72 h, spleen weights in the MCAO + PBS group (****p < 0.0001) and the MCAO + LIF (**p < 0.01) were significantly lower than the sham group. There was a trend towards a higher spleen weight in the MCAO + LIF group compared to the MCAO + PBS group, but this trend was not significant (*p* = 0.1016). *n* = 18–23 animals per treatment group

Western blotting was used to determine whether MCAO and LIF treatment altered levels of LIFR in the spleen at 72 h post-MCAO. At 72 h post-MCAO, there was a significant change in splenic LIFR expression among sham, PBS, and LIF-treated rats (*F*_2,21_ = 3.511; *p* = 0.0484). LIFR levels in the spleens of LIF-treated (*p* = 0.0086) but not PBS-treated rats were significantly lower than those of sham rats (Fig. 1a). When the levels of LIFR were normalized to the weight of each spleen, there was no significant difference in normalized splenic LIFR levels between sham-operated, PBS-treated, and LIF-treated rats (*H* = 4.295; *p* = 0.1168). However, there was a trend towards increased normalized LIFR levels in PBS-treated rats compared to the other treatment groups (Fig. 1b). Compared to spleens from sham-operated rats, MCAO and LIF treatment caused a significant overall change in the average spleen weight (*p* = 0.0008, *F*_2,56_ = 8.143). There was a significant decrease in spleen weight among rats treated with PBS after MCAO compared to the sham-operated rats (*p* = 0.0001). Rats treated with LIF after MCAO had significantly smaller spleen weights compared to sham-operated rats (*p* = 0.0062). There was a trend towards larger spleens among rats treated with LIF post-MCAO compared to the PBS-treated group, but this trend was not significant (*p* = 0.1016; Fig. 1c).

### Splenocytes express the LIF receptor after MCAO and LIF treatment

**Fig. 2:**
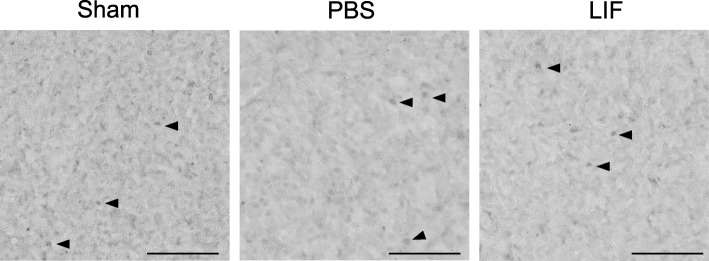
LIFR+ splenocytes are observed in the spleen after MCAO and LIF treatment. DAB immunohistochemistry was used to visualize the expression of LIFR among splenocytes in tissue from sham-operated, PBS-treated, and LIF-treated cells. Although LIFR+ splenocytes were found in all three treatment groups, LIFR+ positive cells showed punctate immunoreactivity while tissue from PBS- and LIF-treated animals showed more diffuse immunoreactivity. Arrows identify representative cells. Scale bar = 50 μm

Spleen tissue sections were stained with antibodies against LIFR in order to visualize the LIF receptor in splenocytes after sham surgery, MCAO + PBS treatment, and MCAO + LIF treatment. Punctate LIFR immunoreactivity was observed in spleens from sham-operated rats while spleens from the MCAO + PBS and MCAO + LIF groups showed more diffuse staining in splenocytes. Arrows are used to identify representative cells (Fig. 2).

### Splenic CD11b expression decreases after MCAO and LIF treatment

**Fig. 3:**
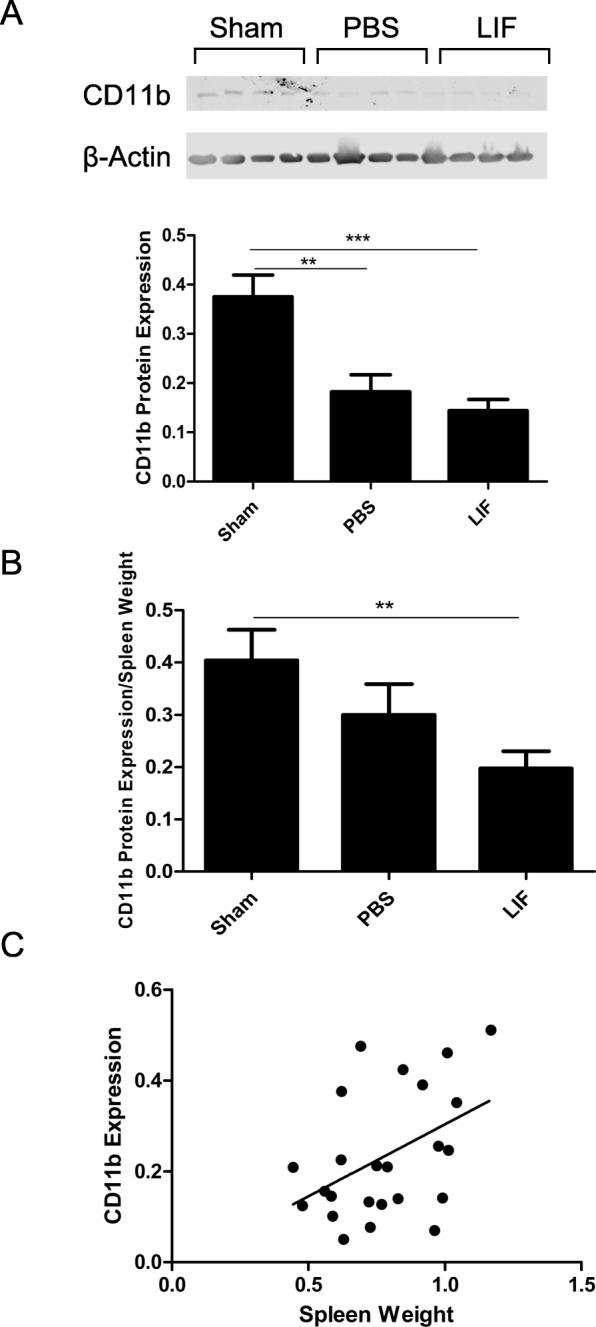
Splenic CD11b levels are significantly decreased after MCAO and LIF treatment. **a** Western blotting was used to measure levels of CD11b, a marker of activated macrophages/microglia, in the spleen 72 h after sham surgery and MCAO. Levels of CD11B were significantly lower in the MCAO + PBS group (**p < 0.01) and the MCAO + LIF (***p < 0.001) were significantly lower than the sham group. **b** When CD11b levels were normalized to spleen weight, there was a significant decrease in normalized CD11b after LIF treatment compared to sham levels (**p < 0.01). **c** According to the results of the Pearson correlation analysis, there was a significant correlation between spleen weights and CD11b levels in the spleen (*p < 0.05). *n* = 8 animals per treatment group

Western blotting was used to measure CD11b protein expression in the spleens of PBS and LIF-treated rats euthanized at 72 h after MCAO. At 72 h, levels of CD11b in spleen tissue were significantly altered after MCAO and LIF treatment (*p* = 0.0003; *F*_2,21_ = 12.51). There was a significant drop in CD11b levels in the spleens of PBS-treated rats (*p* = 0.002) and LIF-treated (*p* = 0.0002) rats compared to those of sham rats (Fig. 3a). When the CD11b levels were normalized to the spleen weights of each sample, there was a significant overall change in normalized CD11b levels (*p* = 0.0335; *F*_2,21_ = 4.009) and a significant decrease in normalized CD11b after MCAO + LIF treatment compared to sham levels (*p* = 0.0043; Fig. 3b). To determine the relationship between spleen weight and levels of CD11b in splenic tissue, Pearson *r* correlation analysis was performed. There was a significant positive correlation between the spleen weight at 72 h post-MCAO and sham procedure and splenic CD11b protein expression (*p* = 0.0149; Pearson *r* = 0.4437; Fig. 3c).

### IL-12 p40 levels in the spleen are not altered after MCAO and LIF treatment

**Fig. 4:**
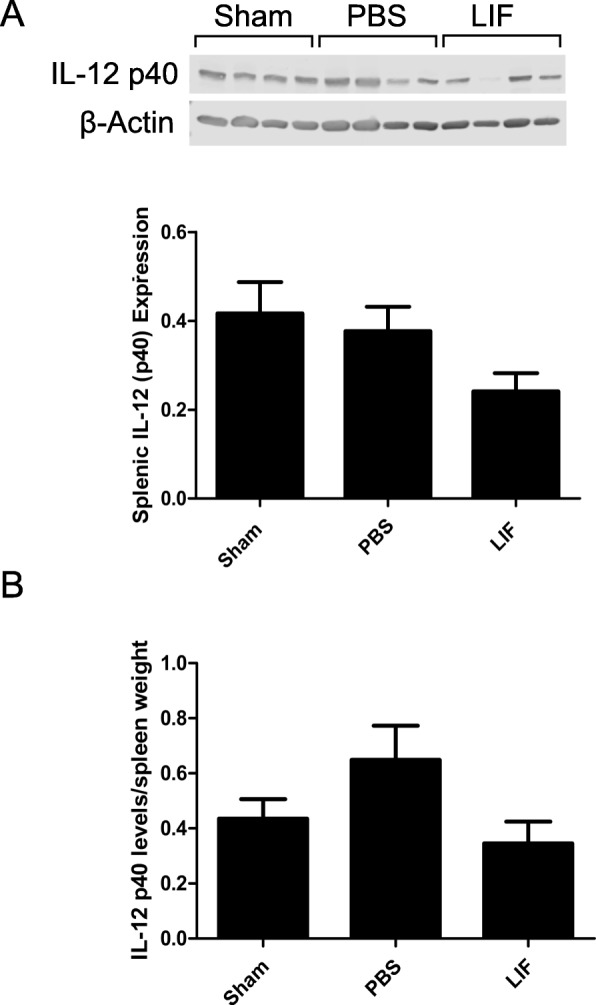
LIF does not alter splenic IL-12 p40 levels after MCAO. Western blotting was used to measure levels of the IL-12 p40 subunit in the spleen 72 h after sham surgery and MCAO. **a** There was no significant overall change in IL-12 p40 levels after MCAO and LIF treatment, but there was a trend towards decreased IL-12 p40 levels in the MCAO + LIF group compared to the sham and MCAO + PBS groups that approached significance. **b** When IL-12 p40 levels were normalized to spleen weights, there was a trend towards increased IL-12 p40 in the MCAO + PBS group compared to the MCAO + LIF and sham groups. *n* = 8 animals per treatment group

To measure release of IL-12 after MCAO and LIF treatment, western blotting was used to detect levels of IL-12 p40 in splenic tissue. There was no overall significant alteration in IL-12 p40 protein levels at 72 h after MCAO and LIF treatment (*p* = 0.098; *F*_2,21_ = 2.600). However, there was a strong trend towards decreased IL-12 p40 levels in the spleens of LIF-treated rats compared to PBS-treated and sham rats (Fig. 4a). When splenic IL-12 p40 levels were normalized to spleen weights, there was a trend towards increased normalized IL-12 p40 levels in the spleens of PBS-treated rats compared to LIF-treated and sham-operated rats (*F*_2,21_ = 2.721, *p* = 0.0890; Fig. 4b).

### TNFα, but not IL-6 and IL-1b, are altered by MCAO + LIF treatment at 72 h after MCAO

**Fig. 5:**
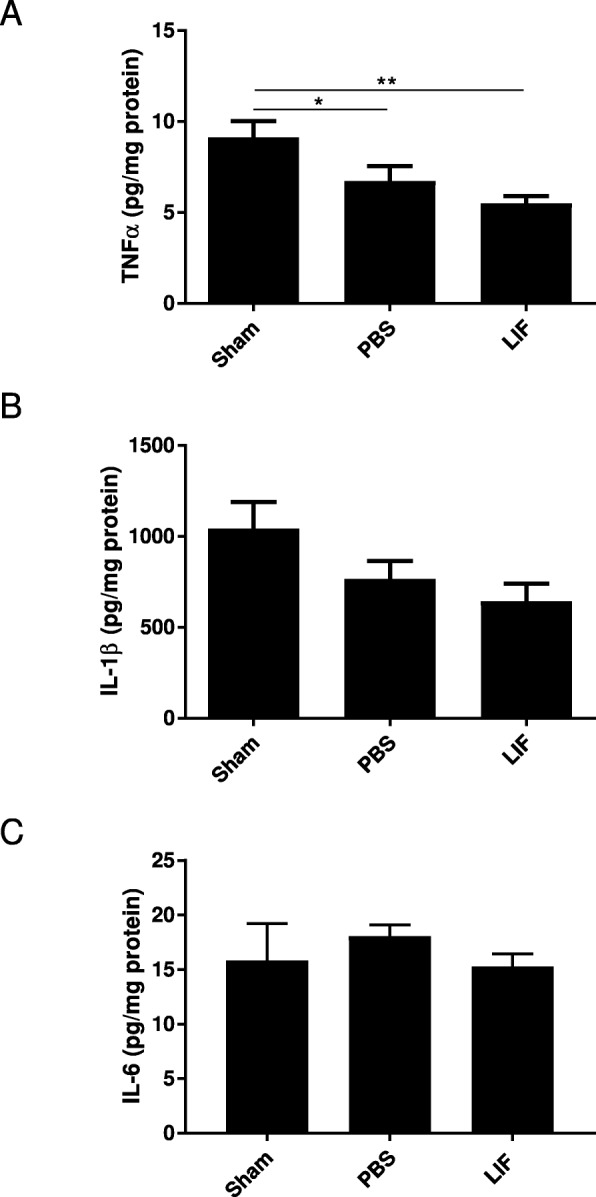
MCAO + LIF treatment alters splenic TNFα, but not IL-1β and IL-6. ELISA was used to measure levels of TNFα, IL-1β, and IL-6 in spleen tissue at 72 h after MCAO. **a** There was a significant decrease in TNFα levels after MCAO + PBS treatment (*p < 0.05) and MCAO + LIF treatment (**p < 0.01) compared to sham levels. However, there was no significant overall change in **b** IL-1β levels or **c** IL-6 levels between treatment groups. *n* = 4–7 animals per treatment group

ELISA was used to measure levels of TNFα in the spleen tissue of sham, PBS-treated, and LIF-treated rats at 72 h after MCAO. There was a significant alteration in TNFα levels at this time point (*F*_2,21_ = 6.181; *p* = 0.0077. TNFα levels were significantly lower after MCAO compared to sham levels (*p* = 0.0347) and after MCAO + LIF treatment compared to sham levels (*p* = 0.0012). However, there was no significant difference between the mean TNFα levels in the MCAO + PBS and MCAO + LIF groups (*p* = 0.1038; Fig. 5a)*.* ELISA was also used to measure levels IL-1β and IL-6 in spleen tissue. There was a trend towards decreased IL-1β (*F*_2,20_ = 3.157; *p* = 0.0644; Fig. 5b) and IL-6 (*F*_2,13_ = 0.3225; *p* = 0.7300; Fig. 5c) levels after MCAO + LIF treatment but this difference was not statistically significant.

### Splenic IL-10 is not altered by MCAO + LIF treatment

**Fig. 6:**
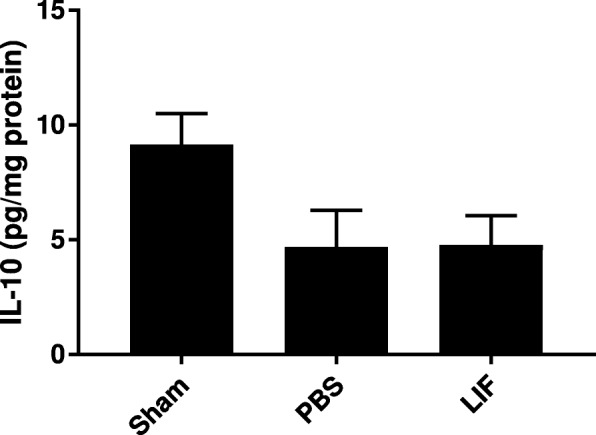
IL-10 levels are not altered by MCAO and LIF treatment. ELISA was used to measure IL-10 in spleen tissue samples. Although there was a trend towards decreased IL-10 after MCAO (regardless of PBS or LIF treatment), the change was not quite statistically significant overall. *n* = 7–8 animals per treatment group

Levels of IL-10 were measured in spleen tissue at 72 h post-MCAO using ELISA. Although there was a trend towards decreased IL-10 levels in the MCAO + PBS and MCAO + LIF groups that approached significance (*F*_2,20_ = 3.437; *p* = 0.0521), the overall difference was not statistically significant (Fig. 6).

### LIF reduces CD3 immunoreactivity in the spleen

**Fig. 7:**
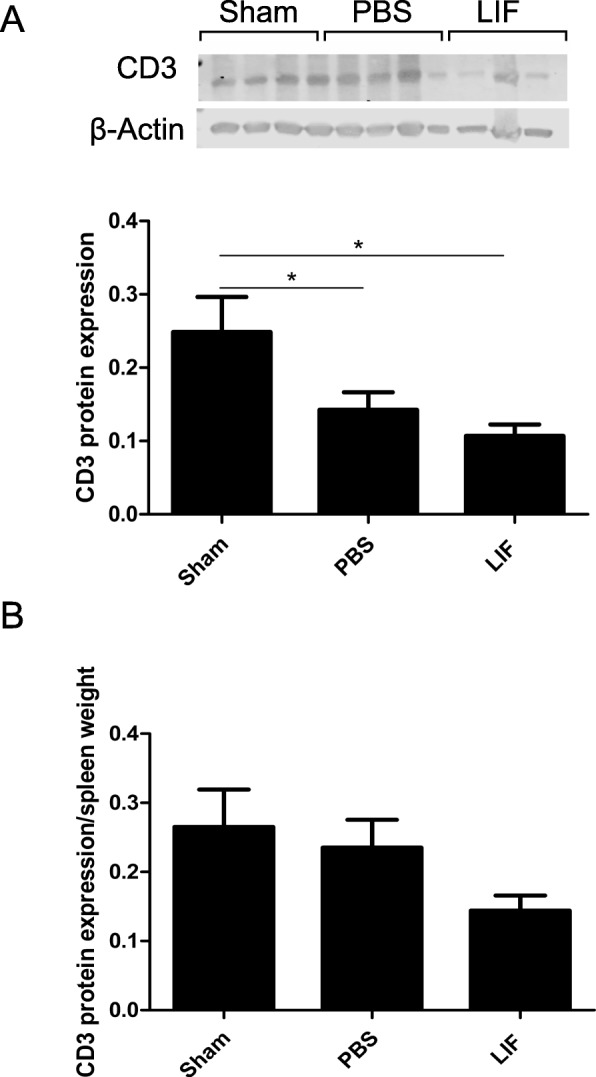
LIF decreases CD3 levels in the spleen after MCAO. Western blotting was used to measure levels of CD3, a pan-T cell marker, in spleen tissue. **a** At 72 h after sham surgery or MCAO, there was a significant decrease in CD3 levels between sham-operated rats and the PBS and LIF treatment groups (*p < 0.05). **b** However, there was no significant change in CD3 expression when protein levels were normalized to spleen weights. *n* = 7–8 animals per treatment group

To determine whether LIF decreases development of mature T cells, western blotting was used to measure levels of CD3, a pan-T cell marker, in spleen tissue at 72 h after MCAO. At 72 h, there was a significant change in CD3 levels between spleen tissue from sham-operated, PBS-treated, and LIF-treated young rats (*F*_2,20_ = 5.198, *p* = 0.0152). There were significant decreases in overall CD3 levels in the spleens of PBS-treated (*p* = 0.0405) and LIF-treated rats compared to sham-operated rats (*p* = 0.0113; Fig. 7a). When CD3 levels were normalized to spleen weight, there was no significant difference in normalized CD3 between the three treatment groups (*F*_2,20_ = 2.434; *p* = 0.1132; Fig. 7b).

### FoxP3+ immunoreactivity is observed in the spleen after MCAO + LIF treatment

**Fig. 8:**
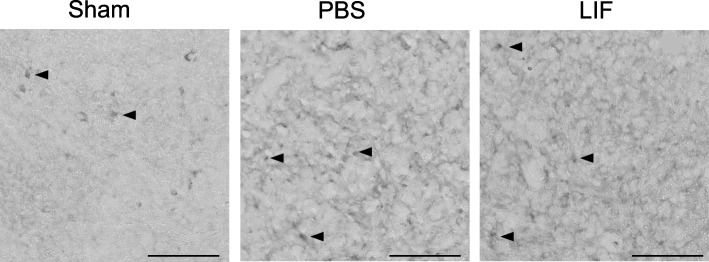
FoxP3+ cells are observed in the spleen after MCAO and LIF treatment. Antibodies against FoxP3 were used to label Tregs in representative spleen tissue sections. Although FoxP3+ cells were found in representative tissue of sham-operated animals, there was noticeably more immunoreactivity for FoxP3 in spleens of MCAO + PBS and MCAO + LIF animals. Arrows identify representative cells. Scale bar = 50 μm

Spleen sections from sham-operated, PBS-treated, and LIF-treated rats were stained with FoxP3 antibodies to identify the presence of Tregs. FoxP3+ cells were found in representative spleen sections from all three treatment groups, although FoxP3 immunoreactivity was more prominent in the representative sections from the MCAO + PBS and MCAO + LIF groups (Fig. 8).

### IFNγ release is decreased by MCAO and LIF treatment

**Fig. 9:**
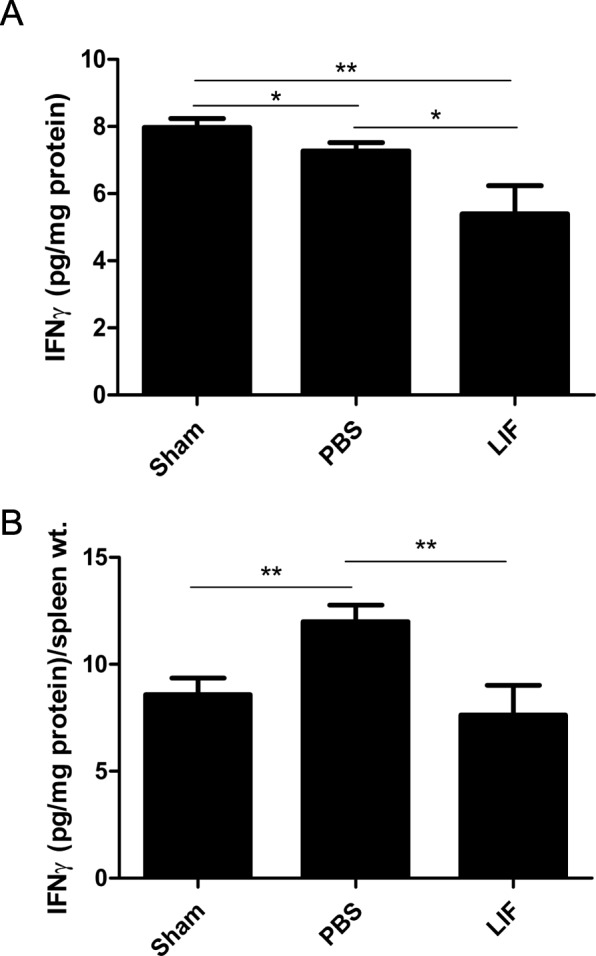
IFNγ release in the spleen decreases after MCAO and LIF treatment. IFNγ release in spleen tissue was measured using an ELISA kit and normalized to the total protein concentration. **a** At 72 h after MCAO or sham surgery, there was a significant decrease in IFNγ release after MCAO + PBS treatment (*p < 0.05) and MCAO + LIF treatment (**p < 0.01) compared to sham levels. There was also a significant decrease in IFNγ release after MCAO + LIF treatment compared to the MCAO + PBS group (*p < 0.05). **b** When IFNγ levels were normalized to spleen weights, normalized IFNγ was significantly higher in the MCAO + PBS group compared to the sham or MCAO + LIF groups. *n* = 8 animals per treatment group

IFNγ levels in spleen tissue from sham, PBS-treated, and LIF-treated rats was measured using an ELISA kit and normalized to the total protein. There was a significant change in IFNγ levels in the spleen tissue of sham-operated, PBS-treated, and LIF-treated rats at 72 h post-MCAO (*F*_2,21_ 6.365; *p* = 0.0069). There was a significant decrease in IFNγ levels in the spleens of the MCAO + PBS group (*p* = 0.0364) and the MCAO + LIF group (*p* = 0.0095) compared to the sham levels. In addition, spleen tissue from the MCAO + LIF group had significantly lower IFNγ levels compared to tissue from the MCAO + PBS rats (*p* = 0.0324; Fig. 9a). Normalizing IFNγ levels in each sample to the spleen weight also showed a significant alteration in normalized splenic IFNγ after MCAO and LIF treatment (*F*_2,21_ = 5.052, *p* = 0.0162). MCAO + PBS treatment significantly increased normalized IFNγ compared to sham levels (*p* = 0.0038), but LIF treatment significantly reduced normalized IFNγ levels compared to PBS treatment (*p* = 0.0080; Fig. 9b).

### LIF treatment counteracts the increase in splenic IP-10 after MCAO

**Fig. 10:**
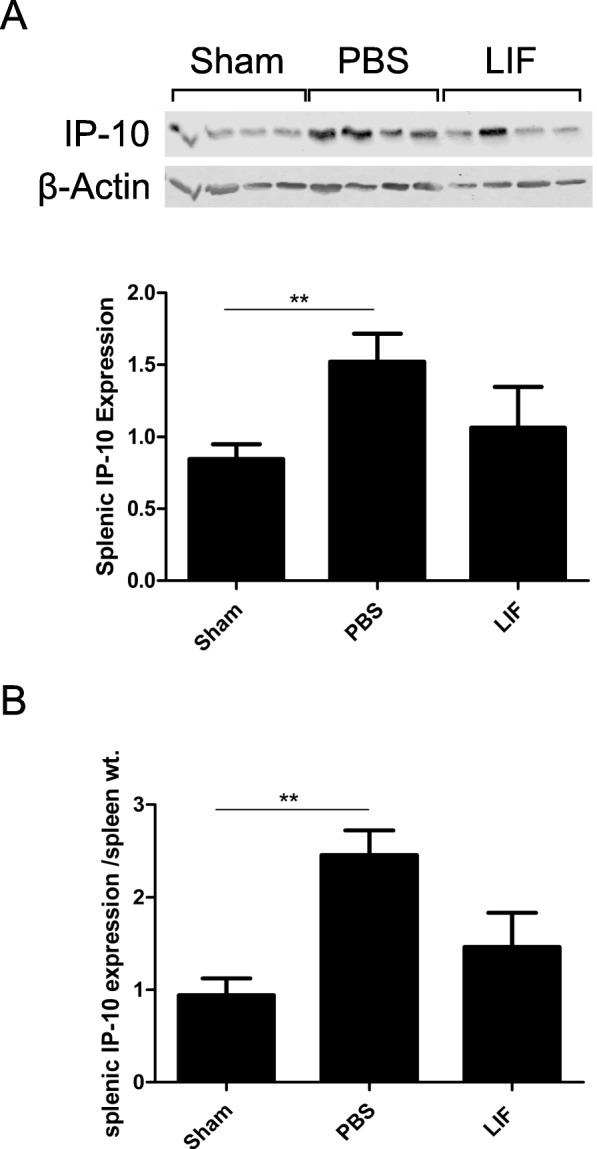
LIF Prevents the upregulation of splenic IP-10 levels at 72 h after MCAO. IP-10 protein levels were measured in spleen tissue using western blot. **a** There was a significant increase in IP-10 levels in the spleens of the MCAO + PBS group compared to the sham group (**p < 0.01). However, levels of IP-10 in the spleens of LIF-treated rats were not significantly different from those in sham levels. Furthermore, there was a trend towards decreased IP-10 levels in spleens of the LIF-treated rats compared to those of the PBS-treated rats. **b** This same pattern of expression was also seen when IP-10 levels were normalized to spleen weights (**p < 0.01). *n* = 8 animals per treatment group

IP-10 levels were measured in splenic tissue using western blotting. At 72 h after MCAO, there was a significant alteration in IP-10 protein expression in spleen tissue (*H* = 7.215; *p* = 0.0271). Splenic IP-10 levels were significantly elevated after MCAO compared to sham levels (*p* = 0.0045). The levels of IP-10 in the spleens of LIF-treated rats were not significantly different from sham levels (*p* = 1.000), but there was a non-significant decrease in IP-10 expression compared to IP-10 expression levels of PBS-treated rats (*p* = 0.1572; Fig. 10a). When the levels of IP-10 in each tissue sample were normalized to the spleen mass, there was a significant change in normalized IP-10 levels at 72 h post-MCAO (*H* = 9.065; *p* = 0.0108). Normalized IP-10 levels remained significantly elevated compared to the sham group (*p* = 0.0015). However, treatment with LIF counteracted this upregulation of IP-10 compared to sham-operated animals (Fig. 10b).

### LIF promotes anti-inflammatory phenotype in LPS/IFNγ-stimulated macrophages

**Fig. 11:**
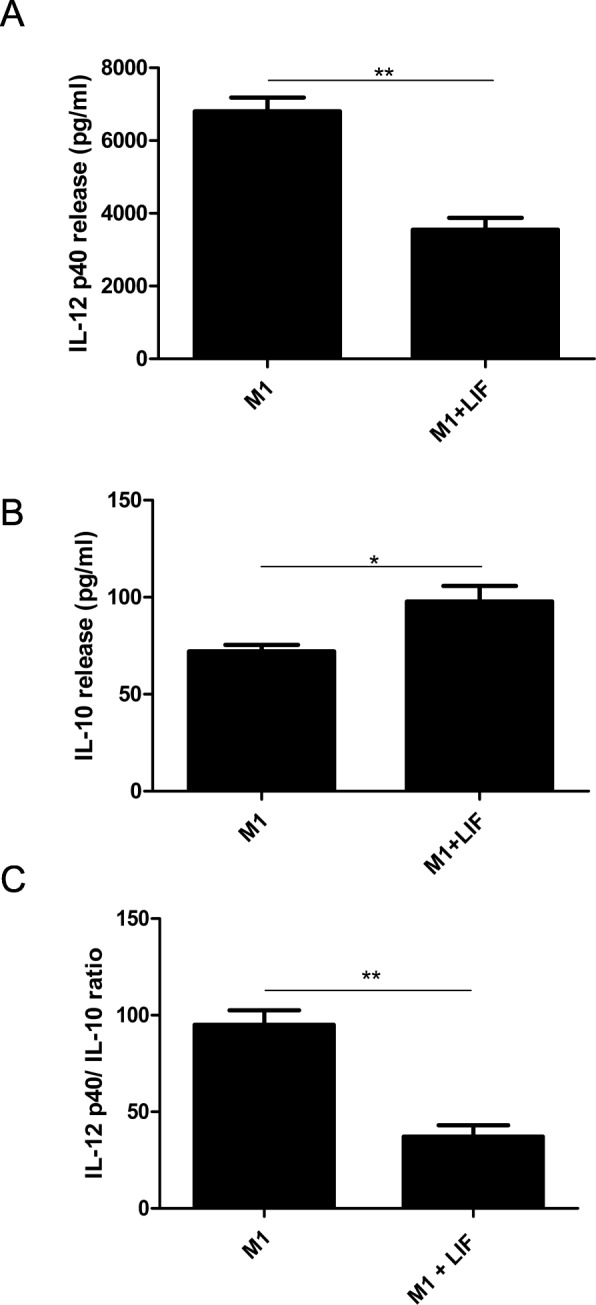
LIF treatment decreases IL-12 p40 release and increases IL-10 release in pro-inflammatory BMDMs. A pro-inflammatory (M1) phenotype was induced in BMDMs via stimulation with IFNγ and LPS. LIF or PBS were co-administered with stimulants. **a** At 24 h after stimulation, macrophage-conditioned media from M1 cells treated with LIF had significantly lower IL-12 p40 release compared to media from M1 cells treated with PBS (**p < 0.01). **b** IL-10 release in macrophage-conditioned media from M1 cells treated with LIF compared to M1 cells treated with PBS (*p < 0.05). **c** The average ratio of IL-12 p40/IL-10 in the macrophage-conditioned media from LIF-treated M1 cells was significantly lower than the IL-12 p40/IL-10 ratio in the PBS-treated M1 cells (**p < 0.01). *n* = 3 wells per treatment group

Prior to stimulation with LPS + IFNγ to induce an M1 phenotype, BMDMs were treated with LIF (20 ng/ml) or PBS. Levels of IL-12 p40 and IL-10 in the supernatant were measured via ELISA. Unstimulated cells treated with LIF or PBS were used as a control. After 24 h, M1 macrophages stimulated with LIF had released significantly less IL-12 p40 compared to M1 cells treated with PBS (*t* = 6.530, df = 4, *p* = 0.0014; Fig. 11a). M1 BMDMs showed significantly elevated release of IL-10 compared to their unstimulated counterparts. Treatment with LIF prior to stimulation further elevated IL-10 release among M1 cells (*t* = 2.942, df = 4, *p* = 0.0212; Fig. 11b). LIF treatment also significantly decreased the ratio of IL-12 p40 release to IL-10 release in the media compared to treatment with PBS (*t* = 6.012, df = 4, *p* = 0.0019; Fig. 11c).

### Activated macrophages/microglia are decreased in the brains of LIF-treated rats

**Fig. 12:**
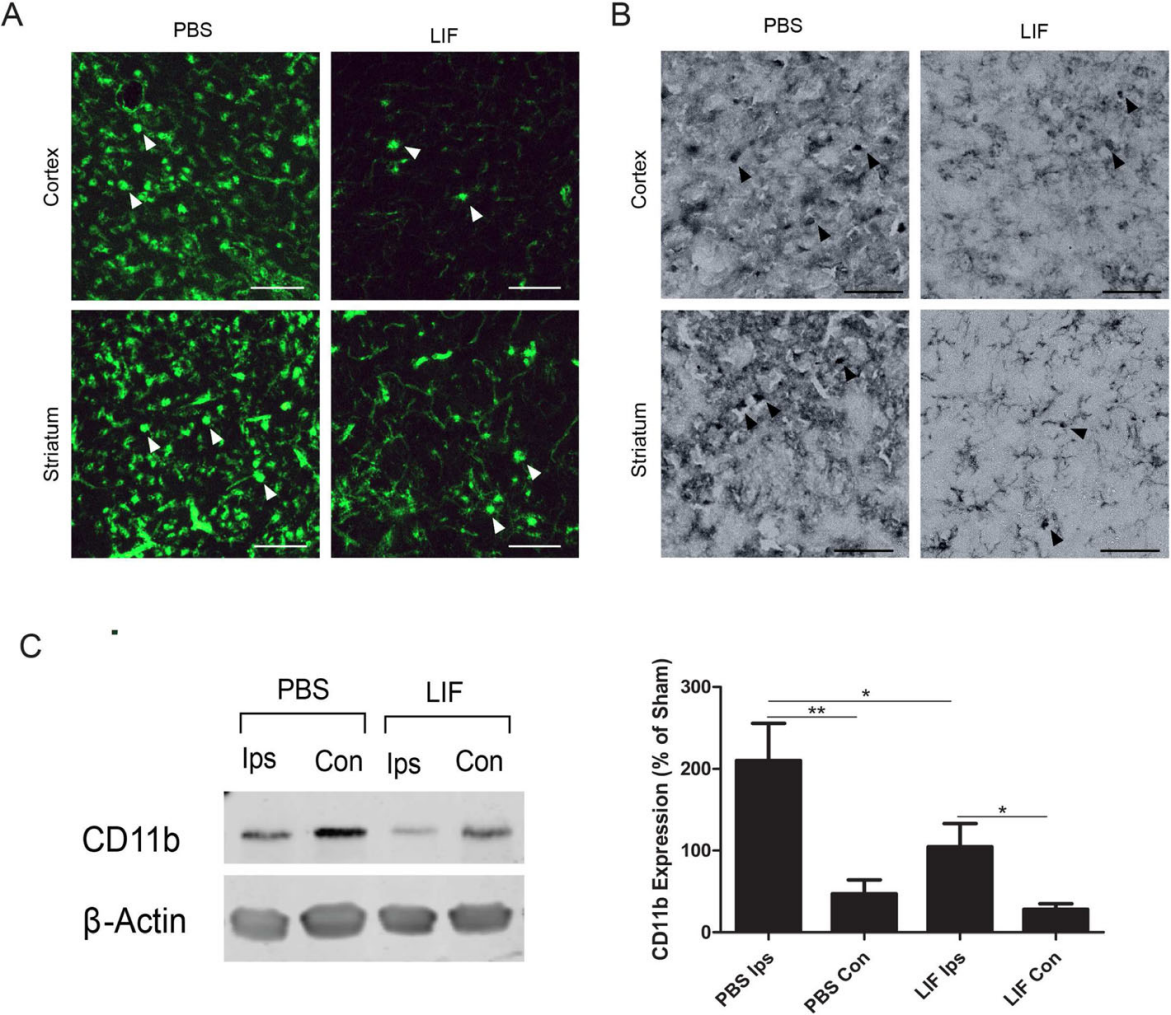
LIF reduces isolectin-tagged fluorescence and CD11b levels in ischemic brain tissue. **a** Ipsilateral tissue sections from the MCAO + LIF treatment group showed less isolectin-tagged fluorescence in the cortex and striatum than tissue from the MCAO + PBS treatment group. Furthermore, isolectin-tagged cells in the striatum displayed a ramified phenotype, while isolectin-tagged cells in the striatum showed an amoeboid phenotype after MCAO + PBS treatment. Arrows indicate representative cells. Scale bar = 100 μm. **b** CD11b antibodies were also used to label cells in the cortical and striatal brain tissue from representative animals. While more amoeboid CD11b + cells were found in the cortex and striatum of brains from representative PBS-treated animals, the CD11b + cells in the brains of LIF-treated animals showed a more ramified phenotype. Scale bar = 50 μm. **c** Western blotting was performed to show levels of CD11b in the ipsilateral and contralateral brain tissue in PBS and LIF-treated rats. There was a significant decrease in the ipsilateral CD11b levels after LIF treatment compared to PBS treatment (*p < 0.05). CD11b levels were significantly elevated in the ipsilateral hemispheres of the PBS-treated (**p < 0.01) and LIF-treated (*p < 0.05) animals compared to their contralateral counterparts. *n* = 5–6 animals per treatment group

Activated macrophages/microglia were visualized in fixed brain tissue from PBS- or LIF-treated rats using isolectin-conjugated to AlexaFluor® 488 dye. At 72 h after MCAO, there was a less isolectin-tagged fluorescence within the cortical and striatal tissue of LIF-treated rats compared to their PBS-treated counterparts. Furthermore, representative images show that lectin-binding cells in the striatum after LIF treatment displayed a more ramified phenotype. By contrast, lectin-binding cells in the striatum displayed an amoeboid phenotype after PBS treatment (Fig. 12a).

Antibodies against CD11b were also used to label brain sections from animals treated with PBS or LIF after MCAO. Representative images show a number of amoeboid CD11b + cells in the cortex and striatum of tissue from animals treated with PBS after MCAO. By contrast, the cortical and striatal tissue from LIF-treated rats contained more CD11b + cells with a ramified phenotype (Fig. 12b).

Western blotting was used to quantify protein levels of CD11b in homogenized brain tissue at 72 h after MCAO. Levels of CD11b in the ipsilateral tissue of PBS- and LIF-treated rats were normalized to the average CD11b levels in sham brains. There was a significant change in CD11b levels in the brain after MCAO and LIF treatment (*F*_3,18_ = 7.800; *p* = 0.0015). CD11b levels were significantly decreased in the ipsilateral hemisphere of LIF-treated rats compared to those of PBS-treated rats at 72 h after MCAO (*p* = 0.0432). Likewise, CD11b levels were significantly higher in the ipsilateral hemisphere of LIF-treated rats (*p* = 0.0301) and PBS-treated rats (*p* = 0.0037) compared to their contralateral counterparts (Fig. 12c).

## Discussion

Although LIF treatment decreased protein levels of CD11b as well as the numbers of isolectin-binding cells, these results indicate that LIF exerts its primary immunomodulatory effects on splenocytes, specifically through the IL-12 p40 /IFNγ/IP-10 axis. Offner et al. previously demonstrated that induction of focal cerebral ischemia in mice stimulates the production of several pro-inflammatory cytokines from splenocytes [60]. This upregulation of cytokine production is followed by a time-dependent decrease in spleen weight due to the migration of splenocyte populations to the ischemic brain [18, 23, 26, 27].

In accordance with previously published studies, spleen size significantly decreased after MCAO compared to sham treatment, LIF-treated rats had a trend towards a larger spleen size at 72 h after MCAO. Furthermore, there was a significant downregulation in splenic LIFR protein expression in the spleens of LIF-treated rats. LIFR is degraded after prolonged stimulation with LIF in many cell types, which indicates that the splenocytes were responsive to peripherally administered LIF after stroke [61]. Although this laboratory has previously shown that the decrease in spleen size is observed as early as 24 h after stroke, this laboratory did not observe any LIF-mediated alteration in LIFR expression or spleen size prior to the 72-h time point [27]. However, published manuscripts from this group have also shown that most prominent effects of LIF are observed at 72 h after MCAO, including improvement in motor skills, decreased tissue damage, and upregulation of antioxidant enzymes [46, 47]. Therefore, it should be expected that the prominent anti-inflammatory effects of LIF are observed at this time point.

Although this study primarily examined the effects of LIF in the spleen and brain after stroke, it is entirely possible that LIF signaling is indirectly modulating the post-stroke immune response through its actions in other tissues. Ajmo et al. previously demonstrated that the activation of adrenoreceptors in the spleen is responsible for the splenic contraction that promotes the migration of immune cells to the brain after stroke [23]. Due to its effects on the hypothalamus-pituitary-adrenal axis [62], it is possible that LIF administration is influencing the post-stroke immune response through the regulation of cortisol release [62, 63]. Furthermore, leukocytes within the peripheral circulation, including T cells, are responsible for perpetuating inflammation in rodent models of ischemic stroke [60]. Therefore, it is highly likely that systemically administered LIF is also acting on immune cells within the peripheral blood in addition to the spleen.

IL-12 is a heterodimer consisting of two subunits: the 35-kDa p35 subunit and the 40-kDa p40 submit, which is a component of the pro-inflammatory cytokine IL-23. Microglia and perivascular macrophages in brain produce IL-12/IL-23 in response to stimulation with other pro-inflammatory cytokines or damage-associated molecular patterns [19]. IL-12 p40 release from macrophages/microglia promotes further production of IL-12/IL-23 by microglia/ macrophages and perivascular dendritic cells. After the breakdown of the blood-brain barrier due to matrix metalloproteinase and cytokine production by microglia, IL-12 p40-stimulated CD8+ cytotoxic T cells, CD4+ type 1 helper T (Th1) cells, and NK cells release IFNγ [64–66]. The results of the in vitro experiments with BMDMs demonstrate that LIF directly reduces IL-12 p40 release from pro-inflammatory macrophages. Furthermore, LIF treatment promotes in vitro release of IL-10 in BMDMs, an anti-inflammatory cytokine that counteracts IL-12 p40-mediated production of IFNγ [67].

While this upregulation of IL-10 was not observed in spleen tissue, it is possible that the migration of monocytes/macrophages into the peripheral circulation prior to 72 h after MCAO prevents us from detecting any significant change in IL-12 p40 or IL-10 in the spleen at this time point [20]. LIF treatment also did not significantly alter levels of IL-1β or IL-6, which are released primarily by monocytes/macrophages [68, 69], at this time point. However, IFNγ was significantly reduced in the spleen after LIF treatment, which demonstrates that LIF is primarily influencing the pro-inflammatory signaling generated by splenic T cells [27, 30, 53].

Our lab has demonstrated an essential role for T and NK cell-derived IFNγ in the initiation of the post-stroke splenic response. According to Seifert et al., splenic T cells upregulate IFNγ at 24 h post-MCAO. Increased IFNγ immunoreactivity in the ipsilateral hemisphere at 72 h post-MCAO corresponds with the infiltration of splenic leukocytes. A splenectomy performed 2 weeks prior to stroke onset or the administration of antibodies to neutralize IFNγ significantly reduced neurodegeneration at 96 h post-MCAO. However, administration of exogenous IFNγ restored the neuroinflammatory response in animals that underwent splenectomy [30, 53]. Following release by NK cells and T cells, IFNγ induces the production of several chemokines in macrophages/microglia, including monocyte induced by IFNγ, interferon-inducible T cell α-chemoattractant, and IP-10 [70]. Western blot analysis showed that LIF treatment significantly reduces levels of CD3, a marker for T cells, in the spleen at 72 h post-MCAO. Although CD3 is expressed by all T cells, this reduction coupled with the downregulation in IFNγ production suggest that LIF prevents the maturation of CD8+ cytotoxic T cells, which are the major producer of IFNγ after stroke [71, 72]. FoxP3-labeled spleen tissue from PBS-treated rats did not show a notable difference in immunoreactivity between PBS- and LIF-treated animals. While these results do not rule out a possible effect on CD4 + CD25 + FoxP3+ Tregs, the anti-inflammatory properties of LIF are more likely due to its abilities to suppress IFNγ production by CD8+ and CD4+ Th1 cells.

**Fig. 13:**
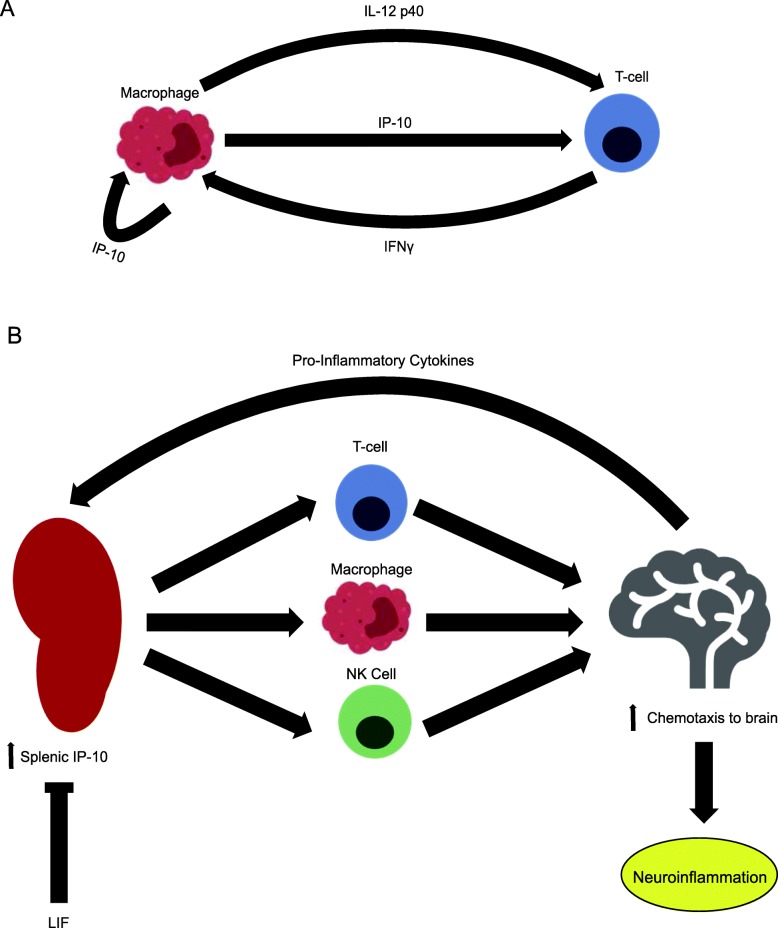
The immunomodulatory effects of LIF. **a** Release of IL-12 from monocytes/macrophages activates the IL-12/IFNγ/IP-10 axis, **b** which promotes chemotaxis to the ischemic brain and increases neuroinflammation. By preventing the increase in splenic IP-10, LIF attenuates the migration of immune cells from the spleen to the ischemic brain

IP-10 is a chemokine that facilitates chemotaxis of pro-inflammatory CD4+ T cells to the ischemic brain via binding to CXCR3 [73]. Alternatively, IP-10, along with other IFNγ-inducible chemokines, promotes post-stroke inflammation via antagonism of the binds to the CCR3 receptor on anti-inflammatory CD4+ T cells [74]. Offner et al. first reported that IP-10 mRNA levels were increased at 22 h after transient MCAO in mice, and Seifert et al. confirmed that the IFNγ/IP-10 axis drives the migration of T cells to the brain after stroke [18, 30, 75]. In this study, splenic IP-10 levels increased after MCAO in PBS-treated rats compared to sham-operated rats, but LIF treatment prevented the upregulation of IP-10 after MCAO. By counteracting the IFNγ-mediated increase in splenic IP-10, LIF treatment would decrease the number of pro-inflammatory immune cells migrating to the brain after stroke (Fig. 13).

Although most of the anti-inflammatory signaling promoted by LIF is occurring in the spleen, the decreased isolectin-tagged fluorescence and normalized CD11b levels in brain tissue demonstrate that LIF either decreases infiltration of monocytes/macrophages into the ischemic hemisphere or attenuates the activation of microglial cells after stroke [76]. According to Leonardo et al., the increase lectin-tagged fluorescence after permanent MCAO corresponds with increased numbers of CD11b + monocytes/macrophages into the penumbra [20]. However, we also observed that CD11b + cells in the cortical and striatal tissue of LIF-treated rats had more of a ramified phenotype, while the amoeboid phenotype was more prevalent in the tissue from PBS-treated rats. Therefore, it is possible that LIF treatment influences microglial activation in addition to monocytes/macrophages derived from the spleen. Since IP-10 is primarily responsible for facilitating chemotaxis of immune cells towards the ischemic brain, the decrease in the number of isolectin-tagged cells and CD11b levels could correspond to a smaller population of immune cells leaving the spleen [30, 77]. This explanation is further justified by the trend towards an increase in spleen weight observed after LIF treatment.

Previously released publications from our laboratory and other independent groups demonstrated that LIF promotes tissue repair and functional recovery after MCAO through Akt-dependent upregulation of protective antioxidant enzymes in neurons and oligodendrocytes. However, evidence suggests that certain Akt-inducing biological therapeutics prevent the infiltration of monocytes/macrophages and lymphocytes into the ischemic brain in addition to promoting anti-oxidation. Rowe et al. showed that administration of human umbilical cord blood (HUCB) cells at 48 h after MCAO promotes oligodendrocyte survival via the upregulation of peroxiredoxin IV and metallothionein III. In a later study by Shahaduzzaman et al., HUCB treatment after MCAO increased peroxiredoxin V expression in neurons through increased Akt signaling [78]. However, soluble factors released from HUCB cells, which include LIF, also promote anti-inflammatory signaling after stroke [46, 79]. Vendrame et al. demonstrated that intravenously injected HUCB cells migrated to the spleen during MCAO and partially counteracted the splenic immune response after stroke. Splenocytes isolated from rats treated with human umbilical cord blood cells after MCAO showed significantly decreased production of IFNγ after stimulation with concavalin-A [80]. In a subsequent study, human umbilical cord blood cell treatment after MCAO significantly attenuated the migration of pro-inflammatory isolectin-binding monocytes/macrophages into the ischemic brain [20]. Since the PI3K/Akt signaling axis promotes an anti-inflammatory phenotype in microglia/macrophages [81], it is possible that systemic LIF administration reduces inflammation among peripheral leukocytes through is transduction pathway.

## Conclusion

Through this study and previously published manuscripts, this lab has demonstrated that LIF treatment after stroke promotes neuroprotection and recovery through two distinct mechanisms: Akt-dependent upregulation of antioxidant enzymes [46, 47] and modulation of the IL-12 p40/IFNγ/IP-10 signaling pathway. Future studies dictate that these studies need to be replicated in aged rodents of both sexes, since several groups have identified sex-specific and age-dependent differences in stroke pathophysiology, specifically concerning the post-stroke immune response [82–86].

This laboratory is currently performing studies to determine the neuroprotective efficacy and anti-inflammatory action of LIF in aged (18 month) male and female rats after stroke. Preliminary results show that LIF exhibits potent anti-inflammatory signaling in the splenocytes of aged female rats after stroke. This study on aged animals also includes the use of flow cytometry to determine whether ischemic conditions alter the expression of LIFR on subpopulations of immune cells after stroke [87]. Although there are antagonists available for LIFR [88], it is very difficult to isolate the specific effects of LIFR after exogenous LIF treatment, since LIFR is also crucial for the signaling of other IL-6 family cytokines [89–91]. Therefore, examining expression of LIFR among leukocyte populations might provide further insight into how LIF promotes anti-inflammatory signaling in splenocytes and peripheral blood leukocyte after stroke.

Nevertheless, these data demonstrate the need for new therapeutics that target the peripheral immune response in addition to directly promoting neuroprotection after stroke.

## Abbreviations

BMDM: Bone marrow-derived macrophage
ELVO: Emergent large vessel occlusion
IFNγ: Interferon gamma
IP-10: Interferon Inducible Protein 10
LIF: Leukemia inhibitory factor
MCAO: Middle cerebral artery occlusion
NK: Natural killer
Th1: Type 1 helper T cells
Th17: IL-17+ helper T cells
tPA: Tissue plasminogen activator
Tregs: Regulatory T cells
